# Chitosan from Virgin and SFE-Spent Fungi as a Raw Material for Disinfecting Gels

**DOI:** 10.3390/polym17243315

**Published:** 2025-12-16

**Authors:** Maria-Beatrice Coltelli, Marco Santin, Giulio Panicucci, Andrea Lazzeri, Stefano De Trovato, Simone Arca, Emilio D’Alessandro, Daniele Pietra, Francesca Cartoni, Antonella Castagna

**Affiliations:** 1 Department of Civil and Industrial Engineering, University of Pisa, Via Diotisalvi 2, 56122 Pisa, Italy; andrea.lazzeri@unipi.it (A.L.); francesca.cartoni@unipi.it (F.C.); 2 Department of Agriculture, Food and Environment, University of Pisa, Via del Borghetto 80, 56124 Pisa, Italy; marco.santin@unipi.it (M.S.); g.panicucci15@studenti.unipi.it (G.P.); antonella.castagna@unipi.it (A.C.); 3 L.O.M. Petrolchimici s.r.l., Via Dorsale 11/18, 54100 Massa, Italy; s.detrovato@lompetrolchimici.it; 4 RDPower s.r.l., Strada delle Campore 11/13, 05100 Terni, Italy; arca@rdpower.it (S.A.); dalessandro@rdpower.it (E.D.); 5 Alidans s.r.l., Via Vecchializia, 48–Pontasserchio, 56017 San Giuliano, Italy; pietra@alidans.com

**Keywords:** fungi, mushroom, chitin, chitosan, shrimps, supercritical fluid extraction, supercritical CO_2_

## Abstract

The valorization of extraction residues from biomass waste through a cascade approach contributes significantly to promote circular economy practices and facilitates the transition toward more sustainable functional materials, like chitosan. Virgin and spent fungal biomass, previously subjected to supercritical fluid extraction (SFE) using CO_2_, was further processed through demineralization and deproteinization to isolate chitin. This chitin was then deacetylated to obtain chitosan, and the yield of each step was evaluated. Although the extraction process requires further optimization, all the samples were characterized using infrared spectroscopy to assess compositional changes resulting from the treatments and compared with commercial counterparts. Chitosan solutions in acidic water were used to formulate hydroalcoholic gels, with ethanol pretreatment enabling compatibility between chitosan and alcohol. This study highlights the potential of chitosan—sourced from shrimps or fungi—as a sustainable raw material for disinfecting-gel applications, offering promising insights into its role in polymer-based formulations.

## 1. Introduction

**Figure 1:**
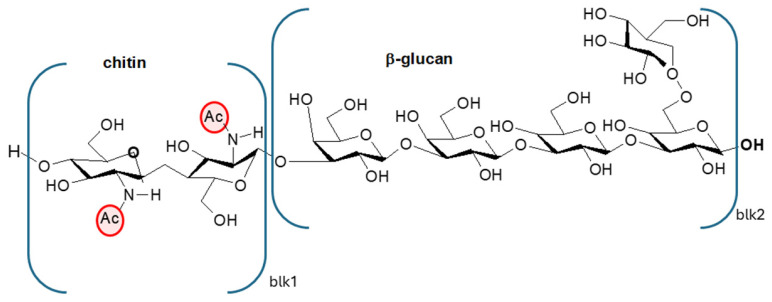
Chitin linked to a β-glucan macromolecule typical of fungi. Ac = acetyl group.

The valorization of industrial byproducts represents a valuable strategy to reduce environmental impact while generating economic benefits, as it enables the recovery of materials that would otherwise be discarded [1]. Chitin and chitosan are well-known polymers traditionally derived from food industry byproducts [2], especially from the shells of crustaceans, which may contain up to 40% chitin by weight [3,4]. However, limitations associated with crustacean-derived chitosan, including its seasonal availability, environmental impact, and allergenic potential, together with the growing demand for vegan-friendly materials, have encouraged researchers and producers to explore alternative sources. Among these, fungal biomass has emerged as a promising and sustainable option [5]. Given the growing demand for vegan-friendly materials, the availability of a sustainable alternative source of chitosan from fungi facilitates the development of its production [6,7,8]. Moreover, fungi can be cultivated under controlled and reproducible conditions, enabling continuous and consistent yields and uniform product quality [9,10]. In addition, due to the absence of the allergenic protein tropomyosin, present in crustacean shells [11], fungus-derived chitosan is a safer material for biomedical and food-related uses. Moreover, fungal chitosan possesses distinctive physicochemical characteristics that can be fine-tuned for specific applications. First of all, in several fungal species, if enzymes that can partially convert chitin to chitosan (chitin deacetylases) are present [12] then chitosan can also be present. Moreover, fungal chitin macromolecules contain glucan branches (Figure 1 [13], and this results in a biopolymer with a lower density of amidic groups [4] than those in the ones from crustaceans or insects [14,15]; hence, the deacetylation of this chitin results in a less positively charged chitosan, which is less hydrophilic than the one from crustaceans [16]. This difference can be exploited in the preparation of liquid [17] and solid [18,19] functional coatings for biobased films and sheets, modulating the hydrophobicity/hydrophilicity with respect to characteristics of the substrate. Chitin content is, however, lower in fungi as compared to that in crustacean sources, where chitin and calcium salts are structural elements of the exoskeleton [3,4,20].

Considerable variability in fungal chitin content has been documented, influenced by the taxonomic diversity (fungal family, genus, and species), environmental conditions, growth medium composition, fungal structure examined (mycelium, fruiting body, stipe, etc.), and developmental stage [21,22,23]. While some species belonging to the phylum Mucoromycota contain significant amounts of chitosan within their cell walls, due to the activity of chitin deacetylase, which converts chitin to chitosan during growth, little or no chitosan is present in most Basidiomycota and Ascomycota, except for a few species [24].

Sun-dried or artificial-UV-exposed mushrooms are a promising dietary source of vitamin D, since ergosterol, a triterpene sterol present in fungal cell membranes, upon exposure to UV radiation, is converted to vitamin D_2_ (ergocalciferol) [25]. Mushrooms enriched with vitamin D represent the only non-animal food source that provides significant amounts of bioavailable vitamin D.

*Agaricus bisporus*, a Basidiomycota species, is one of the most widely cultivated and studied mushrooms for the production of vitamin D_2_ [26]. It is also among the most economically important edible mushrooms due to its high nutritional value [27]. From this mushroom, chitin can be extracted, as reported by Hassinia et al. [9] and, successively, converted to chitosan by deacetylation, as reported by Sousa et al. [8]. Currently, *Agaricus bisporus* is often used as a starting source for obtaining vitamin D_2_ via extraction methods [28,29,30], and there is increasing evidence that large quantities of residue or waste biomass from this process may become available in the future.

**Figure 2:**
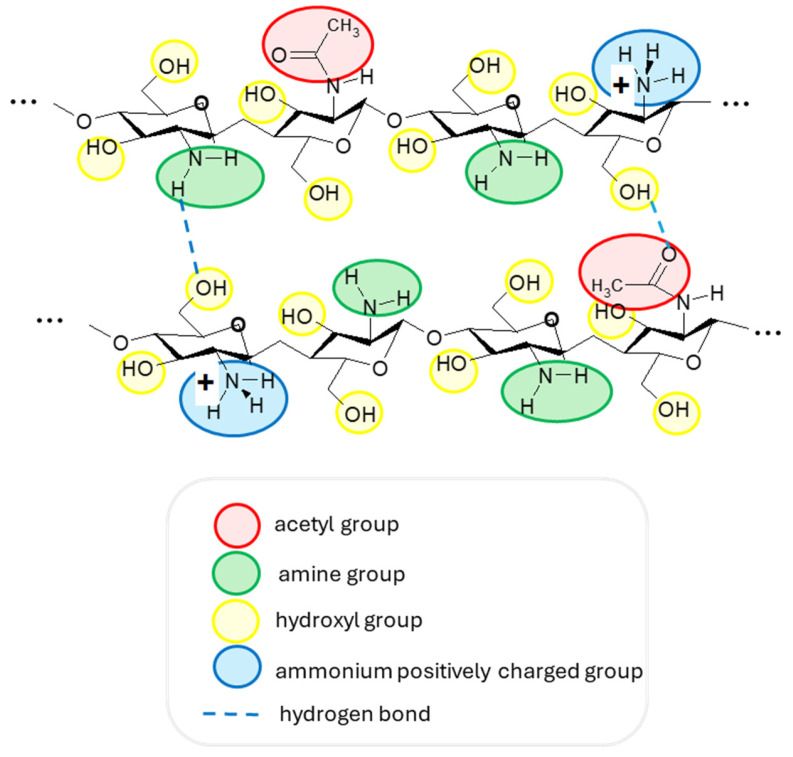
The structure of two chitosan macromolecular fragments with their functional groups and examples of intermolecular hydrogen bonds. A slightly acidic medium is necessary to have protonated ammonium groups with a positive charge.

Chitosan is a linear aminopolysaccharide and a copolymer of two monomers: N-acetyl-D-glucosamine and D-glucosamine, linked by (1→4)-glycosidic bonds. While its precursor, chitin, is predominantly N-acetyl-D-glucosamine, chitosan is mainly D-glucosamine due to the deacetylation process [14]. Thus, chitosan, having amine, amide, and hydroxyl groups (Figure 2), has the advantage of forming intermolecular hydrogen bonds and can be protonated in a slightly acidic solution, resulting in one of the main cationic polymers in nature (pKa = 6.5) [31,32]. In addition, due to its biocompatibility and non-toxic effect, it is used, among other things, in medical applications. Among natural antimicrobial compounds, chitosan is widely used in cosmetic applications, as it can encapsulate active ingredients in various products of industrial interest. Furthermore, due to its fungicidal properties and viscosity, this material is used in creams, shampoos, and lotions [33,34].

Its role as a thickener in water formulations is evident because chitosan allows the viscosities of water-based acidic formulations showing antibacterial, antibiofilm, and antifungal properties to be increased [35]. Gel-based hand sanitizers are important products based on the well-known disinfectant properties of ethanol [36]. Using chitosan and ethanol in combination is, thus, a good idea, as considered in a few papers [37,38,39]. In fact, chitosan is not soluble in ethanol. Hence, hydroalcoholic formulations, typical of many biocidal products, are not suitable for chitosan, which generally precipitates in these solutions. Other biopolymers, like carboxy methyl cellulose (CMC), were used by Zhu et al. [40], whereas other authors used specific biobased compounds, like saponins [37]. The possibility of obtaining disinfectant gels using hydroalcoholic chitosan gel was explored only by Xie et al. [38], but their product, based on commercial chitosan from shrimps, contained silica, which may be deposited on skin when the gel is completely dried. Despite this, it is considered as safe for topical use, as it generally stays on the surface without penetrating the skin barrier [41]. Sensations of dryness on sensitive skin may occur due to its absorbent properties. Moreover, preparing disinfectant gels incorporating chitosan from fungi can be appreciated by vegetarian or vegan consumers [42]. An additional advantage of chitosan–ethanol gels is that once the water and ethanol evaporate, the chitosan remains on the skin surface. Chitosan is known for its intrinsic antimicrobial properties and excellent biocompatibility with skin cells, unlike many conventional antimicrobial agents. These features make such a formulation particularly promising for long-term antimicrobial action that is gentle on the skin.

In line with circular bioeconomy principles, the present study focused on the valorization of residual *A. bisporus* biomass resulting from industrial vitamin D extraction using supercritical CO_2_ extraction by the production of chitosan. Chitin was obtained by demineralization and deproteination, and chitosan was obtained after deacetylation. The materials were chemically characterized in comparison with those extracted from the virgin fungal biomass and with commercial chitosan and chitin obtained from crustaceans to assess differences in terms of structure and film-forming ability. Moreover, the possibility of obtaining disinfectant gels from commercial and fungus-derived chitosan and ethanol was investigated in a comparative way by optimizing the methodology to prepare these gels.

## 2. Materials and Methods

### 2.1. Chitin Extraction and Chitosan Production from Fungal Biomass

The SF biomass resulted from the research activity of LOM Petrolchimici s.r.l. (Massa, Italy), aimed at obtaining D vitamins from non-animal feedstock. The D-vitamin-rich fungi were provided by ALIDANS s.r.l. (San Giuliano, Pisa, Italy). The supercritical CO_2_ extraction was carried out by RDPower s.r.l. (Terni, Italy) at a 5 L SFE plant under 300 bar and 40 °C extraction conditions.

Chitin extraction and subsequent chitosan synthesis were conducted using both virgin (VF) and spent (SF) fungal biomass samples, following the protocol described by Hahn et al. [43], with slight modifications.

To reduce water consumption, the rinsing steps to restore a near-neutral pH before subsequent treatments were performed at room temperature using diluted acidic or basic solutions instead of water.

Initially, the raw biomass underwent desiccation at 50 °C and was ground to a fine, uniform powder.

Demineralization was conducted by treating samples with 0.5 M formic acid solution under continuous magnetic stirring at room temperature for 30 min. Following filtration through non-woven tissues, the material underwent three consecutive washing cycles using dilute NaOH solution (0.0001% by weight) until a neutral pH was achieved. The neutralized samples were then subjected to complete dehydration at 50 °C.

Protein elimination was achieved through alkaline treatment using 2 M sodium hydroxide under stirring conditions at 80 °C for 2 h. The resulting deproteinized material (chitin) was processed through repeated washing cycles with water, followed by thermal drying as described above.

Chitosan synthesis involved the deacetylation of chitin using 12 M NaOH solution with continuous mechanical stirring at 90 °C for 3 h. Subsequently, samples underwent ten washing cycles with 0.0002% by weight HCl solution for neutralization and were then oven-dried.

Finally, a bleaching treatment was conducted using 5% by volume hydrogen peroxide solution at 90 °C for 15 min. Post-treatment samples received three washing cycles with milliQ water for neutralization and underwent final desiccation.

The extraction efficiency of the process was determined by calculating chitin and chitosan yields (%) after each step.

Reference materials were (1) commercial chitosan powder obtained from shrimps, practical grade, Sigma–Aldrich (St. Louis, MI, USA), 417963-100G; (2) commercial chitin powder from shrimps, practical grade, Sigma–Aldrich, C7170-100G; (3) commercial chitosan powder from fungi, GBS24072301.

### 2.2. Materials Characterization

Chitin or chitosan powders were homogenized and ground using a mortar and pestle to reduce the particle size. The resulting powder was then transferred to the ATR crystal for infrared analysis. Spectra were acquired in the 550–4000 cm^−1^ range using a Nicolet 380 FTIR spectrometer (Thermo Fisher Scientific, Waltham, MA, USA) equipped with a Smart iTX ATR accessory featuring a diamond plate. Each spectrum was collected by averaging 256 scans at a resolution of 2 cm^−1^. Spectral data were processed and compared using EZ OMNIC software (OMNIC 7.2, Thermo Fisher Scientific).

For each sample, three spectra were recorded from different aliquots of the powder to ensure reproducibility. The R_AC_ ratio, which correlates with the degree of acetylation [14,44], was calculated using Equation (1) as follows:

(1) $$R_{\mathrm{AC}}=\frac{A_{1620}}{A_{1020}}$$

where A_1620_ is the integrated area of the peak at 1620 cm^−1^ (within the 1695–1618 cm^−1^ range), and A_1020_ is the area of the reference band (1184–1024 cm^−1^). Baseline correction was performed by drawing a line through the minima at approximately 1735 cm^−1^ and 1185 cm^−1^, consistent across all the spectra, using EZ OMNIC software. R_AC_ values were averaged across the three spectra, and standard deviations were calculated for each sample.

Chitosan films were prepared to assess the chitosan’s film-forming properties by casting a solution of chitosan in deionized water containing 3% glacial acetic acid by weight in polystyrene Petri dishes and drying the films at 50 °C for 24 h. The film thickness was in the range 40–80 μm. The films were analyzed under the same ATR-FTIR conditions. Spectra were collected from both sides of each film to assess potential compositional inhomogeneities. In all cases, spectra from opposite sides were found to be identical. Residues left by the prepared hydroalcoholic gels were also analyzed after casting the gel formulations under the same conditions.

The viscosities of the solutions prepared with commercial chitosan from shrimps, commercial chitosan from fungi, and soluble chitosan from VF and SF (1 wt.% chitosan in 1 wt.% acetic acid aqueous solution [45]) were measured at a shear rate of 10 s^−1^ using a rotational viscometer (Lamy Rheology Instruments, Champagne-au-Mont-d’Or, France) equipped with an MK-SV27 spindle. Measurements were performed at 25 °C, in accordance with ASTM D2196. Each determination was replicated three times, and the standard deviation was calculated.

### 2.3. Preparation of Chitosan Hydroalcoholic Gel

Trials related to gel preparations were carried out using chitosan from shrimps (sh) from Aldrich (practical grade) (Table 1). Chitosan (0.4 g) was slowly added to 10 g of water and 0.06 g of acetic acid. The composition was 95.6% water; 3.82% chitosan; 0.57% acetic acid.

In Trial G1, 2 g of this gel was diluted with 8 g of EtOH. In Trial G2, all the different components were then put together, causing the acid concentration to be 0.6% by weight. In Trial G3, 2 g of water, chitosan (0.1 g), and 0.06 g of acetic acid were mixed, and immediately afterward, 2 g of ethanol was added, the components were mixed together, and a very viscous gel was formed. Trial G4 was carried out considering that in the first stage, the dissolution of chitosan (0.065 g) was performed in acidified water (2 g) by adding 0.06 g of acetic acid with a small amount of ethanol (0.4 g). After leaving the gel to homogenize for about 25 min, the rest of the ethanol (8.8 g), in which 0.2 g of glycerol was solubilized, was added. Trials G5 and G6 were carried out using the commercial chitosan from fungi (cf). Trial G7 was carried out using the unbleached chitosan obtained from fungi.

## 3. Results and Discussion

### 3.1. Biomass Recovery from Fungal Samples

Table 2 presents the biomass recovery percentages obtained throughout each processing step for both virgin and spent fungal biomass samples. The demineralization process resulted in comparable recovery rates between the two fungal biomass types, with SF showing only a 2% higher recovery compared to VF samples. Following deproteinization, chitin recovery demonstrated similar values, with VF exhibiting a 6% higher yield than SF samples. Chitin, being a polar polysaccharide, cannot be solubilized or extracted by the supercritical CO_2_ during the processing of the fungal biomass. However, this process, by extracting lipids and other apolar compounds, may induce minor structural changes, such as increased porosity of the biomass matrix, that can facilitate the removal of proteins or water-soluble polysaccharides, resulting in the slightly lower yield observed in the spent fungal biomass. Lam et al. [21] reported variable chitin yields in different forest fungal species, such as *Auricularia auricula-judae*, *Hericium erinaceus*, *Pleurotus ostreatus*, *Tremella fuciformis*, and *Lentinula edodes*, ranging from 2.74 to 55.97%. Similarly, a species-dependent chitin yield is reported, by Irbe et al. [22], in the mycelial biomass of *Heterobasidion annosum*, *Phanerochaete chrysosporium*, *Pleurotus ostreatus*, *Trametes versicolor*, *and Lentinus lepideus* and in the fruiting bodies of *P. ostreatus*, *Agaricus bisporus*, and *Ganoderma applanatum*. Interestingly, though these authors adopted a different extraction procedure, the chitin yield of *A. bisporus* (14.3%) was very similar to that obtained in our study from the virgin biomass (14.5%) of the same fungal species.

Regarding chitosan production, the SF biomass exhibited superior conversion efficiency compared to those of VF samples, with the unbleached chitosan yield being about 33% higher than that obtained from the VF biomass. The probable structural modifications induced by the supercritical CO_2_ extraction used to obtain vitamins from the fungal biomass may have favored the deacetylation step by altering the accessibility and reactivity of the chitin chains. As a consequence, a more efficient deacetylation process may have occurred, as evidenced by the higher chitosan yield obtained from the spent biomass and further confirmed by the higher chitin-to-chitosan conversion efficiency (41%) observed in the SF samples compared to the VF ones. As for chitin, different chitosan yields have been obtained in various forest fungal species, ranging from 2.74% in *T. fuciformis* to 15.67% in *A. auricula-judae* (Lam et al., 2023 [21]). Differences have also been reported in the fruiting bodies of some cultivated species, where chitosan yields were generally very low (0.032–0.054%), except for *A. bisporus*, which provided the highest yield (1.72%; Irbe et al., 2023 [22]), although still lower than that obtained in our study (Table 2).

The yield difference between the spent and virgin fungal matrices became even more evident after the bleaching step, as the SF samples showed a 76% higher chitosan yield compared to the VF biomass (Table 2). Moreover, the bleaching process appeared to be more effective on SF-derived chitosan than on that obtained from VF samples, as indicated by the higher bleached/unbleached CHT yield. This result could also be attributed to a possible lower content of pigments and apolar impurities in the SF sample, thereby reducing the amount of material that needed to be removed during bleaching.

Overall, the extraction and conversion processes resulted in substantial biomass reduction, with the final bleached chitosan representing less than 2% of the initial raw fungal material for both biomass types, though SF consistently demonstrated superior conversion efficiencies across most processing stages. The lower chitin and chitosan yields typically obtained from fungal sources compared to crustaceans can be attributed to fundamental differences in the composition and structure of their respective matrices. In crustaceans, chitin represents the major structural component of the exoskeleton, where it is highly abundant and organized in a dense fibrillar network embedded in a mineralized matrix mainly composed of calcium carbonate and proteins [3]. In contrast, in fungi, chitin occurs as a minor component of the cell wall, as the fungal structural framework is mainly reinforced by β-glucans, mannoproteins, and other polysaccharides, forming a complex matrix that is less enriched in chitin [4,20]. Consequently, the overall chitin content in fungal biomass is significantly lower, and its extraction tends to be less efficient due to the strong interactions with other cell wall constituents and the absence of a discrete chitin-rich layer.

### 3.2. Characterization of Materials

**Figure 3:**
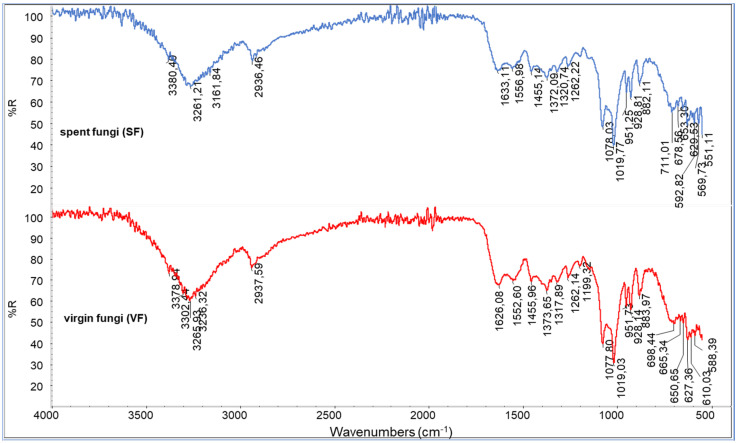
ATR spectra of spent fungal (SF) and virgin fungal (VF) powders.

The infrared spectrum recorded for the powder of the VF shows many characteristic peaks (Figure 3). Mushrooms contain mainly carbohydrates (51.3–62.5%) but also significant amounts of protein (25–35%), dietary fiber (8.0–10.4%), vitamins, minerals, and the majority of the essential amino acids (arginine, leucine, lysine, and tryptophan) [46]. The bands at 3260 cm^−1^ are attributable to -OH stretching, very abundant in polysaccharides. The bands at 1019 and 1078 cm^−1^ are attributable to C-O-C stretching, COH deformation, and COC deformation [47]. The band at about 1630 cm^−1^ can be assigned to amide I (-C=O stretching), typical of chitin. The peaks at about 1550 cm^−1^ can be assigned to C-N stretching and NH deformation (Amide II) and at about 1320 cm^−1^, due to C-N-H deformation (Amide III).

Additional bands at about 1555 cm^−1^ can be attributed to the -NH_2_ deformation typical of chitosan’s repeating units. The well-visible peaks at 1262 (P=O stretching) and 883 cm^−1^ (P-O-P stretching) can be attributed to the presence of polyphosphates. Bands at 2930 cm^−1^ suggest the presence of C-H stretching, which can be attributed to the lipidic fraction of the fungi. Despite the differences in compositions or conditions of cropping [48] that are evident by comparing different fungal species [49], the recorded spectra can be considered as being in good agreement with those reported in the framework of other infrared studies [50,51]. The spectra of the virgin and spent fungi are quite similar. Only slight differences can be seen, suggesting that the content of molecules can be altered by supercritical extraction, even if the variation in concentrations is not significant for appreciating evident changes in the spectra of the SF residue.

**Figure 4:**
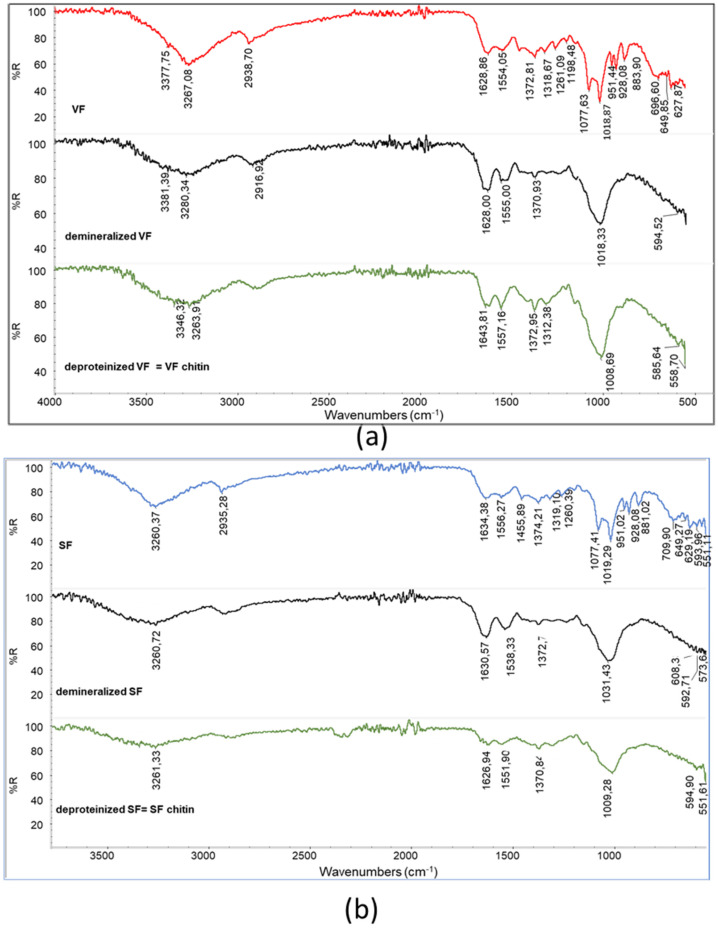
Infrared spectra of (**a**) demineralized VF and VF chitin obtained after deproteination; (**b**) demineralized SF and SF chitin obtained after deproteination.

Demineralization has successfully changed the compositions of the VF and SF, resulting in demineralized samples, from which spectra the peaks attributed to polyphosphates have disappeared (Figure 4). Moreover, the double peak attributable to C-O stretching in VF and SF is no longer present, and only one large main peak can be detected, suggesting that the polysaccharidic fraction was also partially extracted during this step.

The peak shapes are different for the VF (showing a maximum at 1018 cm^−1^) and the SF (showing a maximum at 1033 cm^−1^), suggesting that the effects of demineralization could be slightly different. Interestingly, deproteination leads to samples having the main polysaccharidic band at 1009 cm^−1^. Thus, the removal of protein and other substances soluble in the basic deproteinizing treatment result in a more homogeneous material, having the characteristic peaks of chitin. The chitins obtained from VF and SF are different from the one obtained from shrimps. In order to compare the different samples, R_AC_ was determined for the deproteinized VF and SF as well as for the commercial chitin powder from shrimps (Table 3).

It is evident that for chitin from fungi, the R_AC_ value is significantly lower than the one for chitin from shrimps. Since the obtained value depends on the concentration of amidic groups in chitin, the concentration of these groups is lower in chitin from fungi than in chitin from shrimps. As already explained, chitin in fungi is partially converted to chitosan by enzymes [12]. Moreover, the presence of glucans linked to chitin [4] determines the decrease in the amidic concentration in the sample. Both these differences in the chemical structures of macromolecules can be responsible of the observed lower values.

**Figure 5:**
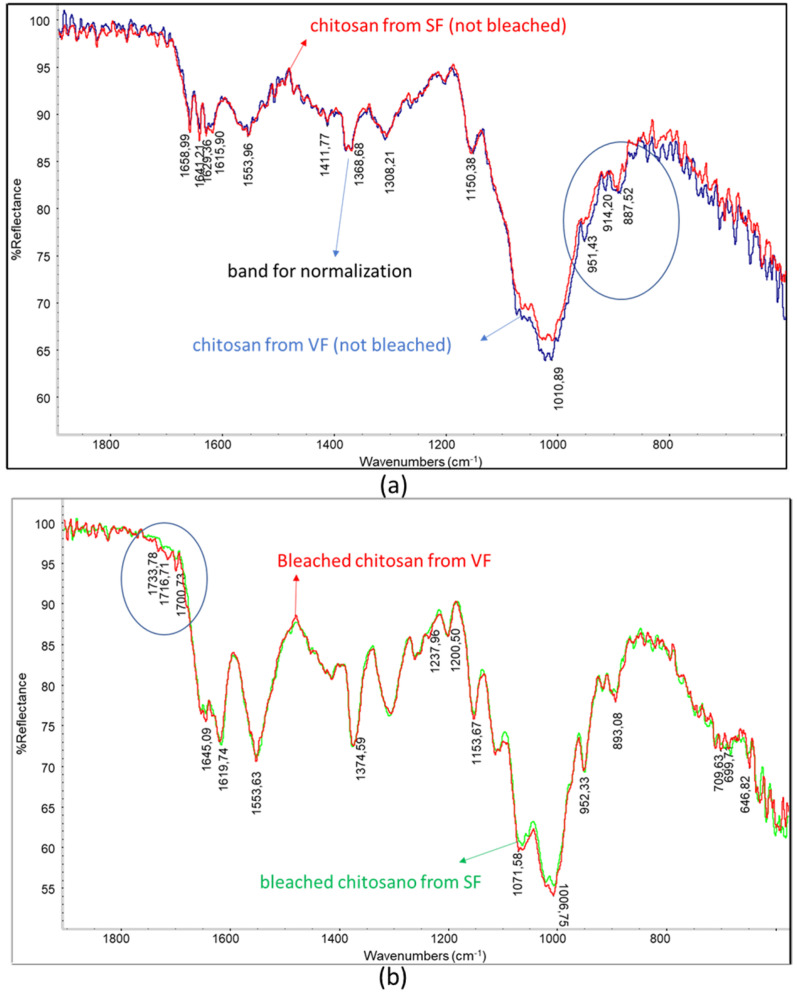
Overlays of infrared spectra: (**a**) chitosan from SF and chitosan from VF; (**b**) bleached chitosan from SF and bleached chitosan from VF. Circles evidence slight changes in the spectrum.

The spectrum for the chitosan obtained from SF shows a slightly lower intensity of the main polysaccharidic band and a higher intensity of the amidic bands than the spectrum for the chitosan from VF (Figure 5a). Interestingly, the bands at 951 cm^−1^ and 915 cm^−1^ can be attributed to the presence of insaturations (out of plane bending), and these bands are more intense in the spectrum for VF chitosan, which was not treated by supercritical extraction (Figure 5a, circled bands). This agrees with the occurrence of the extraction of unsaturated compounds, like vitamins, from the sample thanks to supercritical extraction. The bleached chitosan samples revealed infrared spectra (Figure 5b) different from the one of the unbleached sample (Figure 5a). Bleaching, a treatment that is generally carried out to decolorize chitosan, resulted in significant changes in the polysaccharidic bands. In the spectrum for the chitosan sample, only one peak with a shoulder can be noticed, whereas in the spectra for the bleached chitosan samples, peaks centered at 893, 952, 1007, and 1072 cm^−1^ can be distinguished.

**Figure 6:**
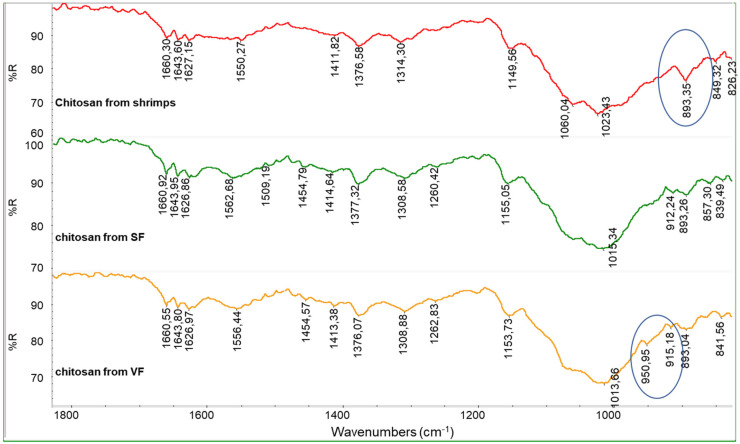
Infrared spectra of chitosan from SF and chitosan from VF (both unbleached) compared with the spectrum of commercial chitosan from shrimps.

It is evident that the spectra of the unbleached chitosan samples are similar to the spectrum of commercial chitosan from shrimps (Figure 6). Interestingly, a peak at 893.5 cm^−1^ can be observed in the spectrum of the chitosan from shrimps, and it can be attributed to the presence of phosphates, which are difficult to fully remove from shrimp-based raw materials.

**Figure 7:**
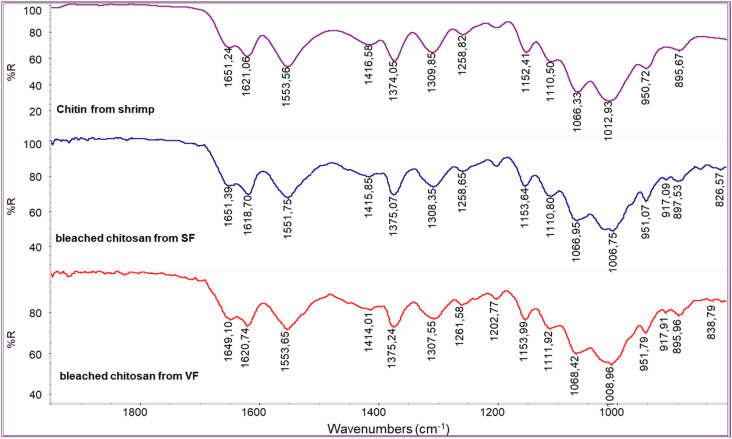
Infrared spectra of bleached chitosan from SF and bleached chitosan from VF compared with the spectrum of commercial chitin from shrimps.

The spectra obtained from bleached chitosan from SF and VF are similar to the one from commercial chitin from shrimps (Figure 7). In this spectrum, the polysaccharidic band has peaks centered at 896, 950, 1012, and 1066 cm^−1^. This is an undesired result because it suggests that the bleaching step converted the chitosan back to chitin. To clarify this, the calculation of R_AC_ was carried out for the chitosan samples (Table 4).

Based on the data presented in Table 4, the R_AC_ values obtained for the chitosans derived from VF and SF are comparable and only marginally lower than those observed for commercial shrimp-derived chitosan. This indicates that the fractionation methodology employed for extracting chitosan from VF and SF was effective. However, as previously reported by Huang et al. [52], the bleaching process led to partial degradation of the chitosan. The investigation revealed that bleaching facilitated the solubilization and removal of the most deacetylated fraction of the chitosan—likely more susceptible to degradation. Consequently, the recovered material exhibited structural characteristics more akin to those of chitin. Moreover, the bleaching step significantly reduced the overall yield due to material loss, rendering the process economically inefficient.

**Figure 8:**
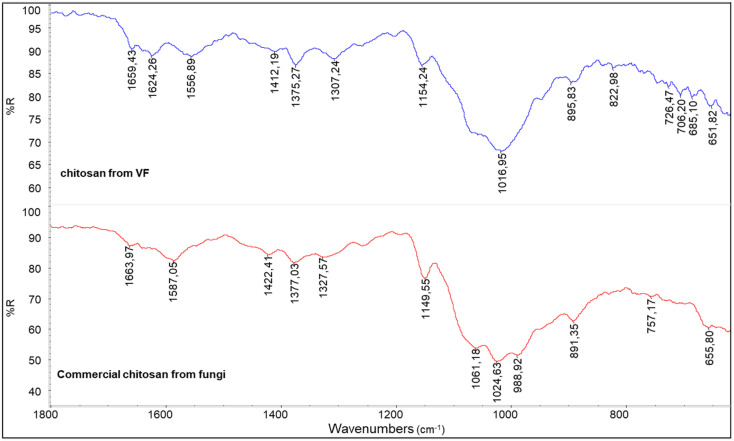
A comparison between the spectra of chitosan from VF and commercial chitosan from fungi.

In comparison with the infrared spectrum of the commercial chitosan powder derived from fungi (Figure 8), that of the chitosan from VF appears generally similar; however, some differences can be attributed to the specific composition of the fungal species used. As previously noted, chitin macromolecules in fungi are often covalently linked to glucan blocks. The presence of these glucans can influence both the shape and intensity of the infrared absorption bands, particularly in the region associated with C-O stretching vibrations. This structural variation likely contributes to the notably low R_AC_ value observed for the commercial fungal chitosan, as reported in Table 4.

The R_AC_ value is lower than the ones obtained for VF or SF chitosan (Table 4), suggesting that the method adopted for the deacetylation of fungal chitin in industry is reasonably more effective.

**Figure 9:**
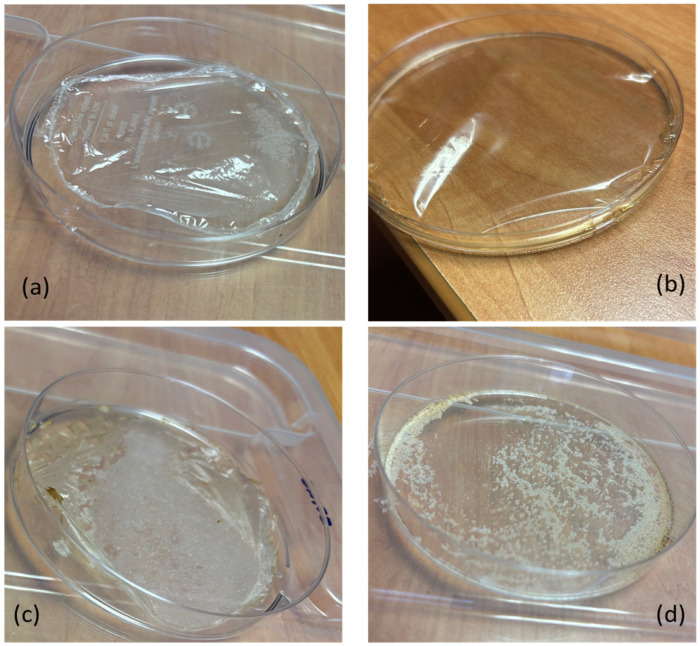
Films obtained by casting chitosan water solution in acetic acid at 3% wt. from (**a**) commercial chitosan from shrimps; (**b**) commercial chitosan from fungi; (**c**) chitosan from SF; (**d**) chitin from SF.

The film-forming properties of chitosan were correlated with several factors, including molecular weight and the ability to form coatings on different substrates [53,54,55]. Therefore, film formability tests were performed to compare the chitins and chitosans obtained from SF and VF with commercial samples (Figure 9). Chitosan samples were dissolved in an aqueous solution containing 3% (*w*/*w*) acetic acid and cast into polystyrene Petri dishes. Films obtained from shrimp-derived chitosan (SH) and commercial fungal chitosan (CF) were transparent, homogeneous, and easily removed from the Petri dish. In contrast, films produced from unbleached chitosans extracted from SF and VF sources were removable but appeared more heterogeneous and discontinuous (Figure 9c). Attempts to form films directly from chitins extracted from SF and VF were unsuccessful, as these samples lacked film-forming properties and could not be detached from the Petri dish. To better understand these differences, solubility tests were performed.

Chitin and chitosan powder samples were dissolved in water acidified with 3 wt.% acetic acid. The undissolved fractions were removed by filtration, dried, and weighed. Residual percentages were approximately 50% for chitins derived from SF and VF, less than 20% for chitosans from the same sources, and negligible for commercial shrimp and fungal chitosans (Table 5). These findings indicate that chitosan exhibits greater solubility under acidic conditions compared to chitin. However, the presence of residual material suggests that impurities or structural heterogeneities may hinder complete dissolution. This behavior correlates with the distinct visual characteristics of the films obtained via casting (Figure 9). Infrared spectroscopy of the residues from SF and VF chitosan samples revealed the presence of chitin, implying incomplete deacetylation. The persistence of this insoluble fraction is likely due to the limited penetration of the deacetylation process in the chitin matrix, resulting in regions that remain acetylated.

The soluble fractions obtained from this test, corresponding to the chitosan component, were recovered and dried to a constant weight. Viscosity measurements of chitosan solutions in 1% acetic acid (Table 5) revealed that chitosans derived from VF and SF exhibited lower viscosities compared to those of commercial chitosans sourced from fungi or shrimps. This suggests that the molecular weight of the chitosan produced through the process described in this study is likely lower than that of typical commercial chitosan. However, it is important to note that viscosity is also influenced by the degree of deacetylation. Specifically, Wang and Xu [56] reported that chitosan solution viscosity increases with higher deacetylation degrees. As indicated by the results in Table 4, the deacetylation of VF and SF chitosans appears to be less effective, which may at least partially explain the reduced viscosities observed in their solutions.

### 3.3. Preparation of Hydroalcoholic Gels

**Figure 10:**
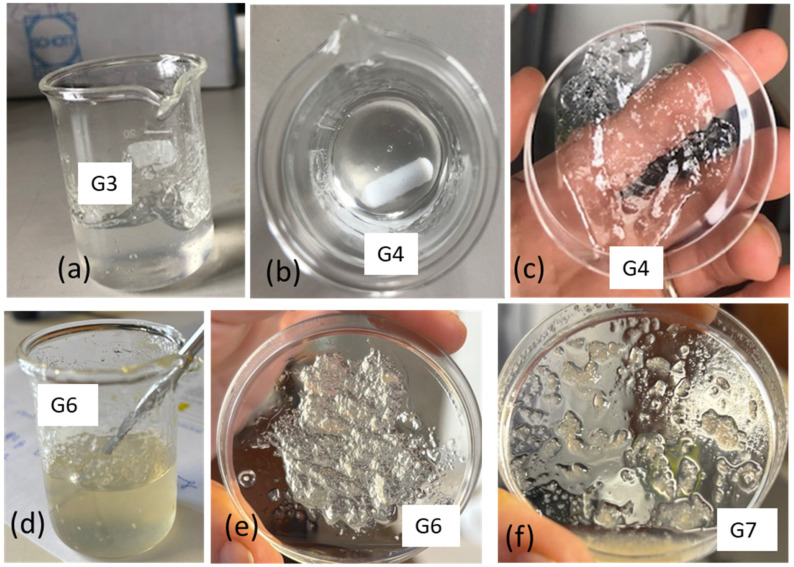
Preparation of gels: (**a**) gel obtained in trial G3; (**b**,**c**) gel obtained in trial G4 from commercial shrimps; (**d**,**e**) gel obtained in trial G6 from commercial fungal chitosan; (**f**) gel obtained using chitosan from SF.

Hydroalcoholic gels were prepared by first dissolving chitosan in water acidified with 3 wt.% acetic acid to form a hydrogel. In a subsequent step, ethanol was added to obtain a formulation predominantly based on ethanol, resembling common disinfectant gels available on the market. In Trial G1, 2 g of this gel was diluted with 8 g of EtOH. The gel did not homogenize well with EtOH, since it tended to form large agglomerates that floated in ethanol. It was thought that this was essentially due to the lower overall acidity, as the acetic acid was also very diluted. In Trial G2, all the different components were then put together, causing the acid concentration to be 0.6% by weight. However, it has been found that chitosan is not soluble in this acidic ethanol/water mixture. The chitosan grains tended to settle on the bottom. In Trial G3, 2 g of water, chitosan (0.1 g), 0.06 g of acetic acid, and 2 g of ethanol were mixed together, and a very viscous gel was formed. After gradually adding the next 6 g of ethanol, this component was incorporated into the gel, resulting in a homogeneous gel with a slightly yellow transparent color, due to the natural color of the chitosan. The obtained aqueous gel was transparent and very viscous (Figure 10a). The solubilization of chitosan in an acidic aqueous solution containing ethanol enables the resulting gel to incorporate additional ethanol in a subsequent step. This noteworthy behavior can be attributed to the formation of hydrogen bonds between chitosan and ethanol during the dissolution and protonation processes in water. Such interactions likely enhance the compatibility between ethanol and the resulting ionomer, which consists of protonated chitosan, with its positive charges balanced by acetate anions.

Trial G4 represents an optimization, where chitosan is minimized, and glycerol is also added. Glycerol, having a high viscosity, can improve this property of the gel [57]. Moreover, glycerol is well known as an additive for lotions and creams that soften the skin [58]. Trial G4 was carried out considering that in the first stage, the dissolution of chitosan (0.065 g) was performed in acidified water (2 g) by adding 0.06 g of acetic acid with a small amount of ethanol (0.4 g). After leaving the gel to homogenize for about 25 min, the rest of the ethanol (8.8 g), in which 0.2 g of glycerol was solubilized, was added. This trial resulted in a transparent and stable gel (Figure 10b,c) and represents an interesting formulation with a minimized content of chitosan.

Trial G5 was carried out like G4 but using the commercial chitosan from fungi (cf). The viscosities of the hydrogel and hydroalcoholic gel were much too low. So, another trial was carried out (G6) using more chitosan (0.1 g). This trial was successful and suggested the possibility of using chitosan from fungi for this application (Figure 10d,e). Trial G7 was carried out using the unbleached chitosan obtained from fungi. The dissolution in acidic water was not complete (Table 5); hence, the obtained gel was less homogeneous and stable than the one obtained using commercial chitosan from fungi (Figure 10f).

## 4. Conclusions

Chitin was successfully extracted from both spent and virgin fungal biomass through a sequential demineralization and deproteination process. Infrared analysis revealed that the resulting fungal chitin exhibited a lower degree of acetylation than chitin obtained from shrimp shells. Subsequent alkaline deacetylation converted chitin to chitosan, with spent fungal biomass yielding approximately 33% more chitosan than virgin fungal biomass. This improved efficiency is likely associated with structural modifications induced by supercritical CO_2_ extraction, which enhanced chitin accessibility and facilitated deacetylation. Consequently, the spent fungi achieved a higher chitin-to-chitosan conversion rate (41%), confirming the potential of valorizing spent fungal biomass within a cascade-processing approach.

Compared to crustaceans, fungi inherently provide lower chitin and chitosan yields due to fundamental differences in cell wall composition. While crustacean exoskeletons contain chitin as the main structural component within a mineralized matrix, fungal cell walls rely primarily on β-glucans and mannoproteins, with chitin forming only a minor fraction. These structural characteristics reduce chitin availability and complicate extraction. Nevertheless, a cascade valorization strategy—where all the components of the fungal biomass are utilized—can offer an efficient and sustainable route for resource recovery.

The bleaching step, although improving purity and appearance, caused substantial material loss, particularly affecting highly deacetylated chitosan fractions. Despite this, chitosan films derived from both virgin and spent fungal biomass displayed satisfactory film-forming abilities, though minor inhomogeneities persisted due to residual chitin particles. Hydroalcoholic gels formulated from commercial shrimp and fungal chitosans were stable and homogeneous, whereas gels produced from the fungal chitosan obtained in this study were less uniform, likely because of incomplete deacetylation and insoluble residues. Overall, these findings underscore the need for further optimization of extraction and deacetylation parameters to produce fungal chitosan with improved solubility, consistency, and suitability for advanced material applications.

## Figures and Tables

**Table 1:** The list of trials carried out for preparing chitosan-based hydroalcoholic gels.

Trial	Deionized Water (g) (wt.%)	Chitosan (g) (wt.%) Type ^a^	Acetic Acid (g) (wt.%)	Glycerol (g) (wt.%)	Ethanol (g) (wt.%)
G1	1.912 (19.1%)	0.0764 (0.76%) sh	0.0114 (0.114%)	**-**	8 (80%)
G2	2 (19.7%)	0.1 (0.98%) sh	0.06 (0.59%)	**-**	8 (80%)
G3	2 (19.7%)	0.1 (0.98%) sh	0.06 (0.59%)	**-**	2 + 6 (80%) ^a^
G4	2 (17.3%)	0.065 (0.56%) sh	0.06 (0.52%)	0.2 (1.7%)	0.4 + 8.8 (80%) ^a^
G5	2 (17.3%)	0.065 cf (0.56%)	0.06 (0.52%)	0.2 (1.7%)	0.4+ 8.8 (80%)
G6	2 (17.3%)	0.1 cf (0.86%)	0.06 (0.52%)	0.2 (1.7%)	0.4+ 8.8 (80%)
G7	2 (17.3%)	0.1 SF (0.86%)	0.06 (0.52%)	0.2 (1.7%)	0.4 + 8.8 (80%)

^a^ sh indicates commercial chitosan from shrimps; cf indicates commercial chitosan from fungi; VF indicates chitosan from virgin fungi (unbleached).

**Table 2:** Biomass recovery (%) after chitin extraction and chitosan production from both virgin fungal (VF) and spent fungal (SF) biomass samples. DM, demineralized biomass; CHT, unbleached chitosan; RAW, raw dried starting biomass.

Fungal Biomass	DM/RAW (%)	Chitin/RAW (%)	CHT/RAW (%)	Bleached CHT/RAW (%)	CHT/Chitin (%)	Bleached CHT/CHT (%)
VF	35.78	14.50	2.60	0.85	17.93	32.57
SF	36.54	13.67	3.46	1.50	25.33	43.22

**Table 3:** Values of R_AC_ determined by calculations performed based on the spectra of the chitin samples.

Material	R_AC_
VF chitin	0.268 ± 0.006
SF chitin	0.217 ± 0.007
Shrimp chitin	0.302 ± 0.007

**Table 4:** R_AC_ data of chitosan materials.

Material	R_AC_
VF chitosan	0.192 ± 0.009
SF chitosan	0.193 ± 0.007
Bleached VF chitosan	0.344 ± 0.007
Bleached SF chitosan	0.351 ± 0.008
Shrimp chitosan	0.167 ± 0.006
Fungal comm. chitosan	0.085 ± 0.008

**Table 5:** Percentages of residues remaining after extraction with acidic water for chitosan and chitin samples, and viscosities of solutions prepared with the soluble fractions.

Material	Residue (wt.%)	Viscosity (mPa·s)
Shrimp chitosan	0	208 ± 6
Fungal comm. chitosan	0	216 ± 5
VF chitosan	17.5	135 ± 4
SF chitosan	13.1	142 ± 3
VF chitin	56.3	n.d.
SF chitin	52.9	n.d.

## Data Availability

The raw data supporting the conclusions of this article will be made available by the authors on request.
